# Relationships of BMI, muscle-to-fat ratio, and handgrip strength-to-BMI ratio to physical fitness in Spanish children and adolescents

**DOI:** 10.1007/s00431-023-04887-4

**Published:** 2023-03-07

**Authors:** Samuel Manzano-Carrasco, Jorge Garcia-Unanue, Eero A. Haapala, Jose Luis Felipe, Leonor Gallardo, Jorge Lopez-Fernandez

**Affiliations:** 1 IGOID Research Group, Department of Physical Activity and Sport Sciences, Faculty of Sport Sciences, Universidad de Castilla-La Mancha, Toledo, Spain; 2 School of Sport Sciences, Universidad Europea de Madrid, Madrid, Spain; 3 Faculty of Sport and Health Sciences, University of Jyväskylä, Jyväskylä, Finland; 4 Institute of Biomedicine, School of Medicine, University of Eastern Finland, Kuopio, Finland

**Keywords:** Body composition, Health, Obesity, Physical activity, Youth

## Abstract

This study aimed to determine the relationship of body mass index (BMI), muscle-to-fat ratio (MFR), and handgrip strength-to-BMI ratio to physical fitness parameters in an active young population according to sex across four different time points. A total of 2256 Spanish children and adolescents (aged 5–18) from rural areas participating in an extracurricular sport in different municipal sports schools participated in this study. Participants were divided into children (5–10 years) and adolescents (11–18 years), boys and girls, and across four different time points (2018, 2019, 2020, 2021). Data on anthropometric measures (BMI, MFR, appendicular skeletal muscle mass) and physical fitness (handgrip strength, cardiorespiratory fitness, and vertical jump) were collected. Boys who were overweight, but especially boys with obesity, had higher absolute handgrip strength in children and adolescents than their normal weight counterparts in 2020 and 2021. Boys and girls with normal weight presented higher cardiorespiratory fitness and vertical jump than their overweight and obese peers over the years. The MFR was directly correlated with the cardiorespiratory fitness and vertical jump variables, but not with handgrip strength, in boys and girls. The handgrip strength-to-BMI ratio in both sexes was positively correlated to the different physical fitness parameters.

*  Conclusion*: BMI, MFR, and handgrip strength-to-BMI can be used as health and physical fitness indicators in this population.

**Table:** 

**What is Known:** *• BMI is the main indicator commonly used as a proxy for obesity for many years. Nevertheless, it cannot differentiate between fat mass and fat-free mass.* *• There are other indicators such as MFR and handgrip strength-to-BMI that might be more accurate and can serve to monitor the health and fitness of children and adolescents.*	
**What is New:** *• MFR showed a positive and significant correlation with cardiorespiratory fitness and vertical jump in both sexes. On the other hand, the handgrip strength-to-BMI presented a positive correlation with cardiorespiratory fitness, vertical jump, and handgrip strength.* *• The use of these indicators obtained through different parameters of body composition and physical fitness can serve as a tool to identify the relationships of the paediatric population with physical fitness.*

**What is Known:**

*• BMI is the main indicator commonly used as a proxy for obesity for many years. Nevertheless, it cannot differentiate between fat mass and fat-free mass.*

*• There are other indicators such as MFR and handgrip strength-to-BMI that might be more accurate and can serve to monitor the health and fitness of children and adolescents.*

**What is New:**

*• MFR showed a positive and significant correlation with cardiorespiratory fitness and vertical jump in both sexes. On the other hand, the handgrip strength-to-BMI presented a positive correlation with cardiorespiratory fitness, vertical jump, and handgrip strength.*

*• The use of these indicators obtained through different parameters of body composition and physical fitness can serve as a tool to identify the relationships of the paediatric population with physical fitness.*

## Introduction

Scientific evidence suggests a progressive increase in obesity and a decrease in physical fitness among children and adolescents worldwide [1, 2]. Excessive time spent on sedentary behaviours and, in particular, sedentary technology use, unhealthy dietary composition, poor physical fitness, and insufficient sleep are the main factors responsible for these public health issues [3–5]. These unhealthy behaviours have gotten worsen due to the restrictions involving SARS-CoV-2 [6]. In this sense, it is necessary to address a greater capacity in policy and adherence towards daily physical activity practice, making visible an existing problem in society and to raise awareness of physical inactivity through better surveillance and monitoring of different parameters.

The Global Action Plan on Physical Activity 2018–2030 (Action 4.2.) [7] and the United Nations’ Sustainable Development Goals [8] suggest monitoring and surveillance of fitness and health to understand the effectiveness of current policies and guide future actions to enhance healthy behaviours among children and adolescents. However, contrary to physical activity surveillance, which is implemented in all countries of the European Union, the monitoring of physical fitness and body composition of children and adolescents is not as extended. Bulgaria, Finland, Portugal, and Slovenia are the main promoters of this type of surveillance [9]. The implementation of field-based fitness test batteries or protocols offers an opportunity to track and record physical fitness parameters such as cardiorespiratory, musculoskeletal, and body composition [10]. But the standardisation of this data into benchmarking health indicators is not that easy. The main indicator commonly used as a proxy for obesity for many years has been the body mass index (BMI) [11]. Nonetheless, it is a relatively poor proxy of body composition in childhood [12] and has been questioned due to its limitations in detecting adiposity in the young population [13]. In addition, it cannot differentiate between fat mass and fat-free mass [14], which may have different effects on health outcomes [15], and it does not inform about the current physical fitness of children and adolescents. Thus, it is important to provide new evidence on other indicators related to body composition and physical fitness that may be more accurate in monitoring and surveillance of health and fitness in children and adolescents.

In recent years, the waist-to-height ratio (WHtR), which combines waist circumference and height, has been used for detecting abdominal obesity [16]. Furthermore, authors have suggested the use of other indicators used less commonly, as they seem to address several of the limitations of BMI, such as the muscle-fat-ratio (MFR) or the handgrip strength-to-BMI ratio. The MFR studies the relationship between skeletal muscle mass and total body fat mass [17, 18]. This indicator relies on precise measurements of body composition and it has been considered the main indicator of low muscle mass [19]. On the other hand, the handgrip strength-to-BMI ratio is calculated as the handgrip strength test result divided by BMI [20, 21]. Due to the fact that handgrip strength can be measured quickly and easily in field-based testing together with BMI, the handgrip strength-to-BMI ratio, which takes into account body composition and fitness parameters, could help to determine the state as well as the evolution of the young population. Additionally, these markers have been correlated with metabolic risk [17, 22], central adiposity [23] in children and adolescents, and arterial hypertension and type 2 diabetes in adults [23–25]. Nonetheless, to the best of our knowledge, the effectiveness of these indicators in relating to body composition and physical fitness among active or partially active children and adolescents is yet to be explored. Therefore, we investigated the relationship of BMI, MFR, and handgrip strength-to-BMI ratio to physical fitness parameters in an active young Spanish population according to sex across four consecutive years.

## Methods

### Participants

**Fig. 1:**
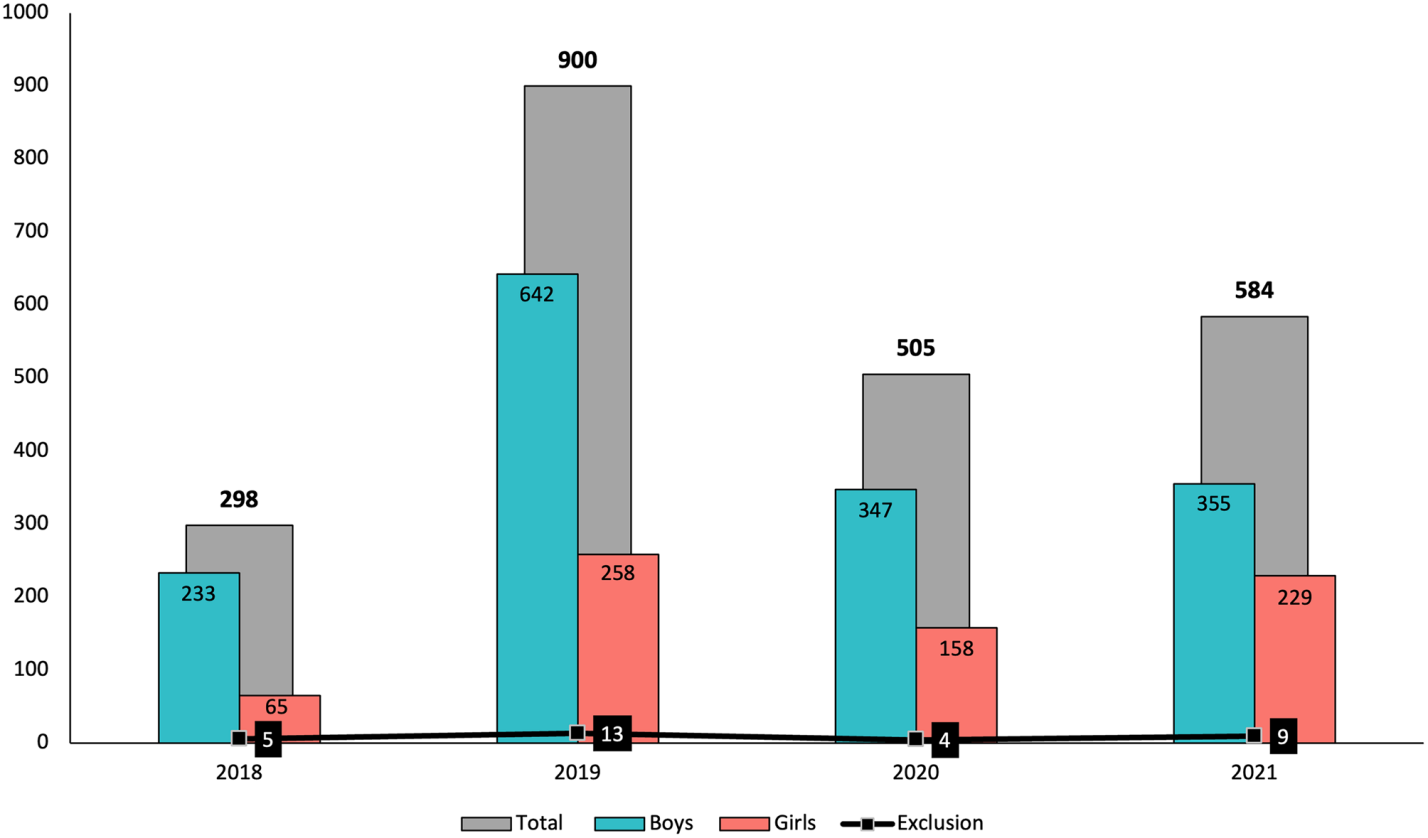
Number of participants in the *Active Health* project over a four-year

Baseline data of the *Active Health* project [26, 27] collected from May 2018 to December 2021 were analysed in this cross-sectional study (Fig. 1). All participants were participating in an extracurricular sport activity at least 2 days a week for a minimum of 1 h each day from different municipal sports schools in Castilla-La Mancha (a central and rural region of Spain). Although in this project all participants enrolled in sports schools are invited to participate, only those who completed all the tests were taken into account in the analysis. A total convenience sample of 2256 children and adolescents aged 5 to 18 years old (11.0 ± 2.7 years; 43.6 ± 15.1 kg; 146.4 ± 16.1 cm) participated in this study. The final sample of the study was formed by 1558 boys (69% of the study population) and 698 girls (31% of the study population). The sample was divided based on sex (boys and girls), age range (children 5–10 years and adolescents 11–18 years, according to other studies) [17], and academic year (2018, 2019, 2020, and 2021). The main exclusion criterion was the presence of physical disability or any health problem which might influence the performance in the fitness tests. Participants’ parents were informed about the aim and nature of the test in the study and written informed consents was obtained. Table 1 presents the descriptive data of the participants (anthropometric and physical fitness variables).

**Table 1:** General characteristics of the sample across 4 years

**Variables**	**2018** **(*n* = 293)**	**2019** **(*n* = 887)**	**2020** **(*n* = 501)**	**2021** **(*n* = 575)**
Age (years)	12.6 (2.12)	10.9 (2.65)	10.9 (2.94)	10.4 (2.58)
Weight (kg)	47.9 (14.07)	42.8 (14.70)	44.6 (16.76)	41.6 (14.21)
Height (cm)	152.7 (13.43)	145.8 (15.77)	146.5 (17.64)	144.2 (15.72)
BMI (kg/m^2^)	20.1 (3.53)	19.6 (3.72)	20.0 (3.94)	19.5 (3.74)
Fat mass (kg)	11.5 (6.01)	10.5 (5.75)	11.2 (6.42)	10.4 (5.79)
Fat mass (%)	23.2 (6.79)	23.6 (6.62)	24.2 (6.70)	24.2 (6.85)
Muscle mass (kg)	34.4 (9.29)	30.6 (9.97)	31.7 (11.39)	29.5 (9.53)
Muscle mass (%)	72.7 (6.41)	72.3 (6.23)	71.7 (6.31)	71.8 (6.47)
ASMM (kg)	1.5 (0.58)	1.4 (0.53)	1.3 (0.49)	1.3 (0.54)
MFR (kg/kg)	3.5 (1.25)	3.5 (1.57)	3.1 (1.13)	3.2 (1.12)
Grip strength-to-BMI (kg/kg/m^2^)	1.3 (0.42)	1.1 (0.40)	1.0 (0.41)	1.0 (0.40)
Handgrip strength (kg)	26.4 (9.20)	20.9 (9.27)	20.0 (9.49)	20.1 (8.70)
Handgrip strength (pc)	64 (27.81)	51 (29.03)	46 (29.21)	56 (28.43)
20-mSRT (stages)	6 (2.15)	5 (2.32)	5 (2.54)	4.5 (2.25)
20-mSRT (pc)	68 (22.05)	67 (24.52)	62 (25.99)	63 (25.06)
VO2max (ml/kg/min)	48.3 (4.97)	48.9 (4.90)	47.8 (5.37)	47.6 (4.65)
Vertical jump (cm)	No data	22.1 (6.48)	23.3 (7.24)	22.0 (6.45)
Vertical jump (pc)	No data	47 (26.61)	44 (25.14)	45 (26.03)

Data are presented as mean (SD)

*kg* kilogrammes, *cm* centimetres, *m* metres, *BMI* body mass index, *ASMM* appendicular skeletal muscle mass, *MFR* muscle-fat-ratio, *pc* percentile, 20-*mSRT* 20-m Shuttle-Run Test, *VO2max* maximal oxygen uptake

This research was carried out in compliance with the standards of the Declaration of Helsinki (2013 revision, Brazil) [28] and following the guidelines of the European Community for Good Clinical Practice (111/3976/88 July 1990) as well as the Spanish legal framework for clinical research on humans (Royal Decree 561/1993 in clinical trials). The *Active Health* project was approved by the Bioethics Committee for Clinical Research of the Virgen de la Salud Hospital in Toledo and by the supervisors of the University of Castilla-La Mancha (Ref.: 508/17042020).

### Assessments

Data collection took place in each of the participating sports schools before and during timetabled extracurricular sports activities on different days. Each test was set up at individual stations and the participants rotated between them in groups of 10–12 every hour, except of the cardiorespiratory fitness test which was done as a group. Each station was controlled by an experienced research. The established protocol of tests explained below:

#### Anthropometric measurements

Each participant underwent an anthropometric assessment utilising a methodology at 5-min intervals, according to prior research [29]. For this assessment, a portable segmental analyser of multifrequency body composition (Tanita MC-780, Tanita Corp., Tokyo, Japan) was used to measure weight (kg), fat mass (kg and %), and muscle mass (kg and %). Height (cm) was assessed with a height rod (Seca 214, Hamburg, Germany). BMI was calculated with the weight (kg) divided by the squared height (m). The appendicular skeletal muscle mass (ASMM) was calculated by the sum of the muscle mass of four limbs, and muscle-to-fat ratio (MFR = ASMM [kg]/fat mass [kg]) was also calculated [30]. The evaluations were conducted while wearing clothing and without shoes.

#### Physical fitness

An adapted version of the extended Assessing Levels of Physical Activity (ALPHA) health-related fitness battery for children and adolescents [10] was used to assess the different parameters of physical fitness. In accordance with earlier studies [31, 32], a percentile (pc) value based on age and sex was used to standardise the findings from all tests. All fitness tests were conducted by researchers, and the order in which they were carried out was as follows:

A handgrip strength with hand dynamometer with adjustable grip was used to evaluate upper-body muscular strength (Constant R Model: 14192-709E). Participants in a full-extension elbow position were required to close their hands with a continuous maximum force for three seconds. The test was performed with the dominant hand and the non-dominant hand alternately. It was possible to try again with a 30-s rest period in between. Each participant’s best score from their dominant hand was taken into consideration for an analysis to the nearest 1 g, and the result was recorded in kilogrammes as absolute values [10]. Pc or relative values based on age and sex were used to standardise the test results [31, 32].

A vertical jump test was completed to assess lower-body muscular power. Height was recorded in centimetres and calculated to the nearest 0.1 cm by photoelectric cells. This technological equipment consists of two parallel bars (Optojump, Microgate, Bolzano, Italy) which measure flight time taken as the duration between take-off and landing. Participants were instructed to jump as high as possible, and three attempts were allowed with 30 s of recovery. The test results were used to standardise as a pc based on age and sex [31, 32].

Finally, cardiorespiratory fitness was assessed by performing a maximum incremental field test (20-m Shuttle-Run Test [20-mSRT]). Participants had to run between two lines 20 m apart while keeping a pace emitted by acoustic signals by a portable speakerphone. The initial speed is 8.5 km h^−1^, which is increased by 0.5 km h^−1^ each min [33]. The test ended when the participant failed to reach the end of the lines concurrent with the audio signals on two consecutive occasions. Otherwise, the test finished when the participant stopped because of fatigue. The results were transformed in stages of 1-min duration, and the maximal oxygen uptake (VO2max) was estimated using the formula by Leger et al. [33]:

VO2max (ml·kg^−1^·min^−1^) = 31.025 + 3.248·*X*_1_ – 3.248·*X*_2_ + 0.1536·*X*_1_·*X*_2_, where the final speed is *X*_1_ (km·h^−1^) and age is *X*_2_ (year as the lower rounded integer). The test was performed only once, and it was performed last so that performance and fatigue did not interfere with the participants.

Lastly, the handgrip strength-to-BMI ratio was estimated with the handgrip strength (kg) and BMI (kg/m^2^).

### Statistical analysis

Data were presented as means ± standard deviations. A Kolmogorov–Smirnov test was used to confirm a normal distribution of the variables. Furthermore, categorical data are presented as absolute and relative frequencies. The dataset is balanced and does not present missing values (except for vertical jump in 2018 data, because this parameter was not evaluated that year). The sample was divided based on sex (boys and girls); age range (children 5–10 years and adolescents 11–18 years, according to other studies) [17]; and year of the analysis (2018, 2019, 2020, and 2021). Differences in physical fitness parameters (i.e. dependent variables) between weight status–based World Health Organization (WHO) BMI-for-age reference (normal weight, overweight, and obese, i.e. independent variable) were evaluated by one-way ANOVA for independent samples due to the categorical format of the factor. In order to evaluate the relationship between physical fitness parameters and anthropometric status based on MFR and handgrip strength-to-BMI, Pearson’s product moment correlation was used due to the scale format of the factor. The level of significance was set at *p* < 0.05.

## Results

### Differences in physical fitness according to BMI

Tables 2 and 3 show the differences between the three weight status–based WHO BMI-for-age references (normal weight, overweight, and obese) in physical fitness parameters separated by sex (boys and girls) and age group. Boys and girls showed significant differences in all physical fitness parameters in the four analysed time points.

**Table 2:** Differences in boys on physical fitness parameters regard weight status–based World Health Organization BMI-for-age reference

		**Boys**											
		**2018**			**2019**			**2020**			**2021**		
		**Normal weight**	**Overweight**	**Obese**	**Normal weight**	**Overweight**	**Obese**	**Normal weight**	**Overweight**	**Obese**	**Normal weight**	**Overweight**	**Obese**
**Handgrip strength (kg)**	**5–10**	17.3 (0.99)	18.0 (1.22)	19.6 (1.51)	14.6 (0.28) ¥	15.7 (0.38)	16.7 (0.42)	13.6 (0.38) ¥	13.4 (0.57)	15.3 (0.52)	14.1 (0.48) Ŷ ¥	17.4 (0.69)	16.3 (0.60)
	**11–18**	28.6 (8.22) Ŷ	32.2 (9.58)	31.2 (10.23)	25.6 (0.46) Ŷ ¥	29.6 (0.63)	29.3 (0.87)	26.9 (0.74)	28.8 (1.08)	30.3 (1.36)	26.7 (0.58)	27.0 (0.96)	27.98 (1.21)
**Handgrip strength (pc)**	**5–10**	58 (6.14)	68 (7.50)	80 (9.28)	47 (57.41) Ŷ ¥	57 (3.24)	63 (3.52)	37 (3.16) ¥	39 (4.70)	55 (4.31)	45 (3.07) Ŷ ¥	68 (4.45)	63 (3.88)
	**11–18**	58 (2.55) Ŷ	73 (3.99)	71 (5.32)	43 (1.93) Ŷ ¥	60 (2.64)	58 (3.68)	44 (2.73) ¥	53 (3.97)	61 (5.01)	56 (2.53)	61 (4.20)	60 (5.34)
**VO2max (ml/kg/min)**	**5–10**	52.0 (0.82) ¥	49.5 (0.99)	46.7 (1.27)	52.4 (0.31) Ŷ ¥	49.9 (0.43) ƛ	46.9 (0.47)	51.5 (0.36) Ŷ ¥	49.4 (0.54) ƛ	46.4 (0.49)	50.6 (0.42) ¥	49.3 (0.53) ƛ	47.0 (0.53)
	**11–18**	50.6 (0.39) Ŷ ¥	48.2 (0.62) ƛ	43.2 (0.85)	50.9 (0.31) Ŷ ¥	48.1 (0.43) ƛ	43.5 (0.60)	51.1 (0.51) Ŷ ¥	46.9 (0.74) ƛ	42.2 (0.93)	49.7 (0.40) Ŷ ¥	46.9 (0.66) ƛ	42.2 (0.85)
**20-mSRT (pc)**	**5–10**	85 (4.48) ¥	72 (5.39)	56 (6.96)	80 (1.76) Ŷ ¥	70 (2.46) ƛ	50 (2.70)	75 (2.23) Ŷ ¥	65 (3.29) ƛ	46 (3.02)	73 (2.50) ¥	65 (3.61) ƛ	50 (3.18)
	**11–18**	70 (1.74) Ŷ ¥	61 (2.74) ƛ	38 (3.76)	69 (1.52) Ŷ ¥	56 (2.09) ƛ	33 (2.93)	70 (2.26) Ŷ ¥	50 (3.31) ƛ	31 (4.14)	65 (1.99) Ŷ ¥	49 (3.31) ƛ	26 (4.24)
**Vertical jump (cm)**	**5–10**	No data available			21.0 (0.44) Ŷ ¥	18.9 (0.60) ƛ	15.9 (0.64)	21.5 (0.39) ¥	20.1 (0.59) ƛ	17.2 (0.54)	21.1 (0.43) ¥	19.8 (0.63) ƛ	17.1 (0.55)
	**11–18**				28.2 (0.57) ¥	27.1 (0.72) ƛ	22.0 (0.91)	30.0 (0.48) ¥	28.6 (0.70) ƛ	25.7 (0.88)	28.3 (0.48) Ŷ ¥	24.5 (0.80) ƛ	21.3 (1.02)
**Vertical jump (pc)**	**5–10**	No data available			62 (2.68) Ŷ ¥	50 (3.64) ƛ	35 (3.95)	62 (2.58) ¥	56 (3.83) ƛ	35 (3.51)	61 (2.79) ¥	50 (4.40) ƛ	37(3.53)
	**11–18**				41 (2.47) ¥	37. (3.13) ƛ	19 (3.94)	40 (2.00) ¥	34 (2.91)	25 (3.67)	44 (2.05) Ŷ ¥	30 (3.40)	19 (4.32)

Data are presented as mean (SD). Ŷ significant differences (*p* < 0.05) between normal weight and overweight. ¥ significant differences (*p* < 0.05) between normal weight and obesity. ƛ significant differences (*p* < 0.05) between overweight and obesity. An one-way ANOVA for independent samples was used as statistical analysis

*kg* kilogrammes, *cm* centimetres, *pc* percentile, *20-mSRT* 20-m Shuttle-Run Test, *VO2max* maximal oxygen uptake

**Table 3:** Differences in girls on physical fitness parameters regard weight status–based World Health Organization BMI-for-age reference

		**Girls**											
		**2018**			**2019**			**2020**			**2021**		
		**Normal weight**	**Overweight**	**Obese**	**Normal weight**	**Overweight**	**Obese**	**Normal weight**	**Overweight**	**Obese**	**Normal weight**	**Overweight**	**Obese**
**Handgrip strength (kg)**	**5–10**	15.2 (0.78)	13.7 (1.29)	No data	13.4 (0.35)	14.3 (5.28)	15.2 (0.72)	12.9 (0.43)	14.4 (0.64)	12.5 (0.80)	12.6 (0.38) Ŷ	14.6 (0.61) Ŷ	13.4 (0.63)
	**11–18**	24.9 (0.81)	25.0 (1.30)	28.7 (1.83)	21.3 (0.50) Ŷ	24.0 (0.71)	24.0 (1.21)	21.1 (0.65)	22.7 (1.07)	12.5 (1.25)	23.4 (0.59)	24.0 (0.86)	17.3 (1.54)
**Handgrip strength (pc)**	**5–10**	61 (7.17)	43 (11.93)	No data	51 (3.49)	62 (5.23)	67 (7.16)	44 (4.47)	63 (6.68)	49 (8.69)	49 (3.43)	59 (5.51)	57 (5.71)
	**11–18**	66 (4.28)	64 (6.87)	81 (9.68)	42 (61.80) Ŷ	62 (4.69)	59 (7.94)	38 (4.01)	50 (6.55)	41 (7.69)	62 (2.96)	66 (4.34)	80 (7.52)
**VO2max (ml/kg/min)**	**5–10**	46.9 (0.63)	46.1 (1.05)	No data	49.7 (0.35) Ŷ ¥	47.6 (0.53)	45.8 (0.71)	48.1 (0.38) ¥	46.6 (0.57)	45.3 (0.74)	48.3 (0.31) ¥	47.1 (0.50)	46.3 (0.52)
	**11–18**	45.9 (0.66) Ŷ ¥	42.2 (1.07)	39.8 (1.51)	46.0 (0.42) Ŷ ¥	43.9 (0.63)	40.0 (1.11)	44.2 (0.62) ¥	41.3 (1.02)	38.7 (1.20)	45.6 (0.54) Ŷ ¥	42.3 (0.78)	39.7 (1.34)
**20-mSRT (pc)**	**5–10**	83 (4.65)	67 (7.74)	No data	83 (2.12) Ŷ ¥	73 (3.22)	58 (4.34)	78 (3.16) Ŷ ¥	64 (4.68)	50 (6.09)	74 (2.76) ¥	65 (4.46)	60 (4.63)
	**11–18**	84 (3.19) Ŷ ¥	65 (5.13)	44 (7.22)	75 (2.55) ¥	65 (3.82)	37 (6.76)	67 (3.94) ¥	52 (6.44)	34 (7.57)	72 (2.72) Ŷ ¥	54 (3.92)	32 (6.76)
**Vertical jump (cm)**	**5–10**	No data available			20.1 (0.46) Ŷ ¥	18.0 (0.64)	15.9 (0.89)	18.7 (0.56) ¥	18.1 (0.34)	15.0 (1.09)	18.8 (0.44) ¥	17.5 (0.71)	16.3 (0.74)
	**11–18**				26.6 (0.67) Ŷ ¥	21.9 (0.96)	19.1 (1.57)	24.6 (0.74) ¥	22.4 (1.19)	19.7 (1.40)	24.3 (0.56) Ŷ ¥	21.2 (0.80)	19.3 (1.38)
**Vertical jump (pc)**	**5–10**	No data available			64 (3.22) Ŷ ¥	50 (4.45)	38 (6.22)	53 (3.56) ¥	48 (5.33)	29 (6.93)	56 (3.10)	48 (5.01)	42 (5.20)
	***11–18***				56 (2.92) Ŷ ¥	35 (4.18)	23 (6.80)	44 (3.56) ¥	33 (5.75)	24 (6.75)	48 (2.80) Ŷ ¥	33 (4.03)	25 (6.95)

Data are presented as mean (SD). Ŷ significant differences (*p* < 0.05) between normal weight and overweight. ¥ significant differences (*p* < 0.05) between normal weight and obesity. ƛ significant differences (*p* < 0.05) between overweight and obesity. An one-way ANOVA for independent samples was used as statistical analysis

*kg* kilogrammes, *cm* centimetres, *pc* percentile, *20-mSRT* 20-m Shuttle-Run Test, *VO2max* maximal oxygen uptake

#### Handgrip strength

In the younger group, boys with overweight showed a higher pc than boys with normal weight in 2019 (*p* = 0.037; ES: 0.24 CI: 0.42 to 19.69) and 2021 (*p* < 0.001; ES: 5.65 to 5.83). Furthermore, boys with obesity had higher handgrip strength (kg and pc) in 2020 (*p* < 0.01; ES: 3.73 to 4.79) and 2021 (*p* < 0.01; ES: 4.15 to 5.13) than boys with normal weight. Finally, in 2021, girls with overweight had higher handgrip strength (kg) than girls with normal weight (*p* = 0.018; ES: 3.99; CI: 0.26 to 3.79).

On the other hand, in the older group, boys with overweight showed higher handgrip strength (kg and pc) than the boys with normal weight in 2018 (*p* < 0.01; ES: 0.39 to 4.36) and 2019 (*p* < 0.001; ES: 7.30 to 7.40). Similarly, boys with obesity had a greater pc than boys with normal weight in 2020 (*p* = 0.009; ES: 4.26; CI: 3.37 to 31.05). In girls, girls with overweight showed higher handgrip strength (kg and pc) than girls with normal weight in 2019 (*p* = 0.01; ES: 0.44 to 4.40).

Finally, boys with obesity had better handgrip strength (kg and pc) than boys with normal weight in both age groups in 2019 (*p* < 0.001; ES: 0.39 to 6.07).

#### Cardiorespiratory fitness

In the younger group, boys with normal weight presented higher VO2max and pc compared to those with overweight in 2018 (*p* < 0.01; ES: 4.15 to 4.57) and obesity in 2021 (*p* < 0.001; ES: 7.57 to 8.14). Likewise, girls with normal weight showed higher VO2max and pc in 2019 (*p* < 0.001; ES: 7.00 to 7.33) and 2021 *(p* < 0.05; ES: 3.75 to 4.55) than girls with obesity. In 2020, girls with normal weight had a higher pc than girls who were overweight (*p* = 0.036; ES: 3.64; CI: 0.73 to 28.35).

In the older group, boys with overweight showed a higher pc than boys with obesity in 2018 (*p* < 0.001; ES: 6.77 to 7.00). Similarly, boys with normal weight had a greater VO2max and pc in 2021 than boys with overweight (*p* < 0.001; ES: 5.13 to 5.74) and obesity *(p* < 0.001; ES: 11.32 to 11.81). In contrast, girls with normal weight had higher VO2max and pc than girls who presented overweight in 2018 (*p* < 0.01; ES: 4.25 to 4.43) and 2021 (*p* < 0.001; ES: 4.91 to 5.73) and obesity in 2018 (*p* < 0.001; ES: 5.32 to 7.14) and 2021 (*p* < 0.001; ES: 5.43 to 7.78). Moreover, in 2019, girls with normal weight had a higher pc than obese girls (*p* < 0.001; ES: 7.42; CI: 20.32 to 55.50) and higher VO2max than girls with overweight (*p* < 0.05; ES: 3.63 to 4.67).

Lastly, in both age groups, boys with normal weight showed a higher VO2max and pc than those who were overweight and obese in 2018 (*p* < 0.001; ES: 4.89 to 11.20), 2019 (*p* < 0.01; ES: 4.58 to 15.47), 2020 (*p* < 0.05; ES: 3.61 to 11.75), and 2021 (*p* < 0.05; ES: 4.33 to 6.20). Girls with the normal weight status had higher VO2max and pc than the girls with obesity in 2020 (*p* < 0.001; ES: 4.82 to 5.95). Finally, boys with overweight had higher VO2max and pc than boys with obesity in 2019 (*p* < 0.001; ES: 5.74 to 8.85) and 2020 (*p* < 0.001; ES: 5.30 to 5.90).

#### Vertical jump

In the younger group, boys with normal weight showed higher vertical jump (cm and pc) than overweight (*p* < 0.05; ES: 3.78 to 3.83) and obese in 2019 (*p* < 0.001; ES: 1.99 to 9.06) and in 2021 in boys (*p* < 0.001; ES: 7.80 to 8.10) and girls (*p* = 0.11; ES: 4.13; CI: 0.44 to 4.59). In 2021, there were no significant differences in the pc of girls (*p* > 0.05).

In the older group, boys with normal weight had higher vertical jump (cm and pc) compared to those with obesity in 2019 (*p* < 0.001; ES: 6.67 to 8.24) and 2021 (*p* < 0.001; ES: 7.38 to 8.74). Also, in 2021, the normal weight group had higher vertical jump (cm and pc) than boys with overweight (*p* < 0.01; ES: 4.96 to 5.74), girls with overweight (*p* < 0.01; ES: 4.26 to 4.48), and girls with obesity (*p* < 0.05; ES: 4.23 to 4.78).

In both groups, boys with overweight showed positively significant differences (cm and pc) than those with obesity in 2019 (*p* < 0.01; ES: 3.89 to 6.26) and 2021(*p* < 0.01; ES: 3.38 to 4.58), except for the pc in the older group (*p* = 0.138; ES: 2.83; CI: -2.23 to 24.30). In addition, boys with normal weight showed higher vertical jump (cm and pc) than those with obesity (*p* < 0.01; ES: 4.91 to 9.04). Similarly, girls with normal weight displayed higher vertical jump (cm and pc) than girls with overweight in 2019 (*p* < 0.05; ES: 3.78 to 5.72) and girls with obesity in 2019 (*p* < 0.001; ES: 5.32 to 6.27) and 2020 (*p* < 0.05; ES: 3.69 to 4.41). Finally, significant differences (cm and pc) were found between boys with overweight and obesity in both age groups (*p* < 0.05; ES: 3.74 to 5.78), except for the pc of the older group in 2020 (*p* = 0.172; ES: 2.69; CI: − 2.35 to 20.23).

### Relationship of MFR and handgrip strength-to-BMI ratio to physical fitness

Sex and age group correlations of anthropometric indicators and physical fitness parameters in the four different time points are presented in Table 4. In both groups of boys, MFR was significantly correlated with cardiorespiratory fitness (*r* = 0.47 to 0.57, *p* < 0.001) and vertical jump (*r* = 0.33 to 0.55, *p* < 0.001). Nevertheless, MFR was not significantly correlated with handgrip strength in the different years (*p* > 0.05). MFR was positively correlated with cardiorespiratory fitness and vertical jump in 2019, 2020, and 2021 (*r* = 0.21 to 0.64, *p* < 0.001) except in the younger group in 2018 (*p* > 0.05). Finally, in girls, MFR was significantly correlated with handgrip strength in the youngest group in 2018 (*r* = 0.65, *p* = 0.011) and in the oldest group in 2019 and 2021 (*r* =  − 0.28 to − 0.23, *p* < 0.05). MFR had no significant correlation with cardiorespiratory fitness and vertical jump in the different years (*p* > 0.05).

**Table 4:** Relationship between physical fitness parameters and anthropometric status based on muscle-fat-ratio and handgrip strength-to-BMI

**Boys**									
		**Muscle-fat-ratio**				**Grip strength-to-BMI**			
		**2018**	**2019**	**2020**	**2021**	**2018**	**2019**	**2020**	**2021**
**Handgrip strength (kg)**	**5–10**	−0.036 (*p* = 0.808)	−0.078 (*p* = 0.210)	0.046 (*p* = 0.560)	−0.046 (*p* = 0.548)	**0.781 (*p* < 0.001)**	**0.713 (*p* < 0.001)**	**0.800 (*p* < 0.001)**	**0.838 (*p* < 0.001)**
	**10–18**	−0.065 (*p* = 0.386)	−0.066 (*p* = 0.204)	−0.002 (*p* = 0.977)	0.142 (*p* = 0.056)	**0.772 (*p* < 0.001)**	**0.786 (*p* < 0.001)**	**0.813 (*p* < 0.001)**	**0.767 (*p* < 0.001)**
**Handgrip strength (pc)**	**5–10**	−0.180 (*p* = 0.227)	−0.040 (*p* = 0.522)	−0.005 (*p* = 0.951)	−0.080 (*p* = 0.301)	**0.659 (*p* < 0.001)**	**0.683 (*p* < 0.001)**	**0.667 (*p* < 0.001)**	**0.665 (*p* < 0.001)**
	**10–18**	−0.085 (*p* = 0.255)	−0.065 (*p* = 0.207)	0.016 (*p* = 0.834)	0.130 (*p* = 0.079)	**0.658 (*p* < 0.001)**	**0.711 (*p* < 0.001)**	**0.714 (*p* < 0.001)**	**0.670 (*p* < 0.001)**
**VO2max (ml/kg/min)**	**5–10**	**0.575 (*p* < 0.001)**	**0.518 (*p* < 0.001)**	**0.569 (*p* < 0.001)**	**0.438 (*p* < 0.001)**	**0.419 (*p* = 0.004)**	**0.442 (*p* < 0.001)**	**0.303 (*p* < 0.001)**	**0.280 (*p* < 0.001)**
	**10–18**	**0.571 (*p* < 0.001)**	**0.477 (*p* < 0.001)**	**0.517 (*p* < 0.001)**	**0.502 (*p* < 0.001)**	**0.503 (*p* < 0.001)**	**0.439 (*p* < 0.001)**	**0.419 (*p* < 0.001)**	**0.419 (*p* < 0.001)**
**20-mSRT** **(pc)**	**5–10**	**0.563 (*p* < 0.001)**	**0.509 (*p* < 0.001)**	**0.532 (*p* < 0.001)**	**0.485 (*p* < 0.001)**	0.291 (*p* = 0.053)	**0.436 (*p* < 0.001)**	**0.330 (*p* < 0.001)**	**0.271 (*p* < 0.001)**
	**10–18**	**0.516 (*p* < 0.001)**	**0.430 (*p* < 0.001)**	**0.533 (*p* < 0.001)**	**0.502 (*p* < 0.001)**	**0.440 (*p* < 0.001)**	**0.415 (*p* < 0.001)**	**0.403 (*p* < 0.001)**	**0.393 (*p* < 0.001)**
**Vertical jump** **(cm)**	**5–10**	**No data available**	**0.486 (*p* < 0.001)**	**0.553 (*p* < 0.001)**	**0.333 (*p* < 0.001)**	**No data available**	**0.487 (*p* < 0.001)**	**0.376 (*p* < 0.001)**	**0.347 (*p* < 0.001)**
	**10–18**		**0.524 (*p* < 0.001)**	**0.448 (*p* < 0.001)**	**0.487 (*p* < 0.001)**		**0.448 (*p* < 0.001)**	**0.452 (*p* < 0.001)**	**0.528 (*p* < 0.001)**
**Vertical jump** **(pc)**	**5–10**		**0.479 (*p* < 0.001)**	**0.531 (*p* < 0.001)**	**0.319 (*p* < 0.001)**		**0.440 (*p* < 0.001)**	**0.329 (*p* < 0.001)**	**0.316 (*p* < 0.001)**
	**10–18**		**0.458 (*p* < 0.001)**	**0.371 (*p* < 0.001)**	**0.411 (*p* < 0.001)**		**0.407 (*p* < 0.001)**	**0.359 (*p* < 0.001)**	**0.427 (*p* < 0.001)**

**Girls**									
		**Muscle-fat-ratio**				**Grip strength-to-BMI**			
		**2018**	**2019**	**2020**	**2021**	**2018**	**2019**	**2020**	**2021**
**Handgrip strength (kg)**	**5–10**	0.437 (*p* = 0.118)	−0.061 (*p* = 0.481)	0.200 (*p* = 0.068)	−0.149 (*p* = 0.103)	**0.860 (*p* < 0.001)**	**0.736 (*p* < 0.001)**	**0.841 (*p* < 0.001)**	**0.726 (*p* < 0.001)**
	**10–18**	−0.002 (*p* = 0.987)	**−0.283 (*p* = 0.002)**	−0.171 (*p* = 0.153)	**−0.231 (*p* = 0.020)**	**0.712 (*p* < 0.001)**	**0.560 (*p* < 0.001**	**0.698 (*p* < 0.001)**	**0.670 (*p* < 0.001)**
**Handgrip strength (pc)**	**5–10**	**0.655 (*p* = 0.011)**	−0.075 (*p* = 0.383)	−0.010 (*p* = 0.930)	−0.138 (*p* = 0.131)	**0.756 (*p* = 0.002)**	**0.666 (*p* < 0.001)**	**0.664 (*p* < 0.001)**	**0.555 (*p* < 0.001)**
	**10–18**	−0.043 (*p* = 0.772)	**−0.248 (*p* = 0.007)**	−0.116 (*p* = 0.337)	**−0.235 (*p* = 0.018)**	**0.574 (*p* < 0.001)**	**0.510 (*p* < 0.001)**	**0.608 (*p* < 0.001)**	**0.534 (*p* < 0.001)**
**VO2max (ml/kg/min)**	**5–10**	0.045 *(p* = 0.879)	**0.235 (*p* = 0.006)**	**0.488 (*p* < 0.001)**	**0.286 (*p* = 0.001)**	−0.206 (*p* = 0.479)	0.142 (*p* = 0.155)	**0.520 (*p* < 0.001)**	**0.374 (*p* < 0.001)**
	**10–18**	**0.599 (*p* < 0.001)**	**0.430 (*p* < 0.001)**	**0.444 (*p* < 0.001)**	**0.449 (*p* < 0.001)**	**0.583 (*p* < 0.001)**	**0.281 (*p* = 0.013)**	0.178 (*p* = 0.143)	**0.415 (*p* < 0.001)**
**20-mSRT** **(pc)**	**5–10**	0.269 *(p* = 0.352)	**0.215 (*p* = 0.012)**	**0.401 (*p* < 0.001)**	**0.234 (*p* = 0.010)**	−0.004 (*p* = 0.990)	0.181 (*p* = 0.071)	**0.443 (*p* < 0.001)**	**0.296 (*p* = 0.001)**
	**10–18**	**0.622 (*p* < 0.001)**	**0.387 (*p* < 0.001)**	**0.461 (*p* < 0.001)**	**0.418 (*p* < 0.001)**	**0.452 (*p* < 0.001)**	**0.218 (*p* = 0.013)**	0.136 (*p* = 0.265)	**0.372 (*p* < 0.001)**
**Vertical jump** **(cm)**	**5–10**	**No data available**	**0.393 (*p* < 0.001)**	**0.439 (*p* < 0.001)**	**0.292 (*p* = 0.001)**	**No data available**	**0.514 (*p* < 0.001)**	**0.500 (*p* < 0.001)**	**0.411 (*p* < 0.001)**
	**10–18**		**0.598 (*p* < 0.001)**	**0.349 (*p* < 0.001)**	**0.501 (*p* < 0.001)**		**0.455 (*p* < 0.001)**	0.316 (*p* = 0.083)	**0.482 (*p* < 0.001)**
**Vertical jump** **(pc)**	**5–10**		**0.388 (*p* < 0.001)**	**0.437 (*p* < 0.001)**	**0.268 (*p* = 0.003)**		**0.429 (*p* < 0.001)**	**0.446 (*p* < 0.001)**	**0.308 (*p* = 0.001)**
	**10–18**		**0.645 (*p* < 0.001)**	**0.326 (*p* < 0.001)**	**0.434 (*p* < 0.001)**		**0.428 (*p* < 0.001)**	0.223 (*p* = 0.066)	**0.340 (*p* = 0.001)**

Values marked in bold are significant. A Pearson product moment correlation was used as statistical analysis

*kg* kilogrammes, *cm* centimetres, *pc* percentile, *20-mSRT* 20-m Shuttle-Run Test, *VO2max* maximal oxygen uptake

On the other hand, in both groups of boys, the handgrip strength-to-BMI ratio was directly correlated with all physical fitness parameters (*r* = 0.27 to 0.83; *p* < 0.001), except with cardiorespiratory fitness (pc) in 2018 (*r* = 0.29, *p* = 0.053). In girls, the handgrip strength-to-BMI ratio was significantly correlated with overall physical fitness parameters in both age groups (*r* = 0.21 to 0.86, *p* < 0.001), except in cardiorespiratory fitness in the younger group in 2018 and in the oldest group in 2020 and in vertical jump in 2020 (*p* > 0.05).

## Discussion

This is the first study investigating the relationship of BMI, MFR, and handgrip strength-to-BMI ratio to muscular strength and cardiorespiratory fitness in physically active children and adolescents according to sex across four consecutive years (2018 to 2021). This study evidenced that weight status taken from BMI as well as MFR and handgrip strength-to-BMI ratio have a significant relationship with different fitness parameters and could be used as health indicators for this population. Our findings showed that children and adolescents with normal weight status, regardless of sex, had higher cardiorespiratory fitness and vertical jump than those who were overweight and obese. In contrast, overweight participants, particularly boys with obesity, displayed significantly higher handgrip strength than those with normal weight status. Moreover, both the MFR and handgrip strength-to-BMI show a significant correlation with cardiorespiratory fitness and vertical jump in both sexes, while handgrip strength-to-BMI also displays a positive correlation with handgrip strength. Even though previous research determined that body composition analyses can allow for the identification and diagnosis of weight status based on BMI, the data of the present study show that the use of other body composition measurements—the MFR and handgrip strength-to-BMI indicators—can serve as a tool for identifying relationships of the paediatric population with physical fitness.

### BMI (weight status) and physical fitness

A total of 57% of participants in the present study had healthy weight status, while 26% and 17% of participants were overweight or obese, respectively. This shows that although the participants regularly practised a sporting activity, approximately 40% of participants had a high BMI value. A recent study investigated the prevalence and incidence of overweight and obesity rates in children and adolescents across eight Spanish regions, suggesting that childhood obesity prevalence and incidence rates vary by region in Spain [34]. In this study, the incidence of obesity in a rural young population descriptively increases over four consecutive years studied, with 2020 being the year where 20% of the total sample were obese. This may be due to the period of confinement caused by COVID-19 as well as their decrease in physical activity and possible worse eating habits during this period according to other studies [35].

An overweight and obese status can lead to a higher prevalence of suffering from metabolic syndrome compared with normal weight status in children and adolescents [36]. In addition, this may have an impact on physical fitness, which has been seen as an important marker of health [37]. Our findings indicate that the association between BMI based on weight status and physical fitness is significant, which extends previous findings in the young population [27, 38, 39]. Furthermore, the association of higher muscular strength with an overweight and obesity status was more pronounced especially in boys. These findings are in accordance with the study by Fernandez et al. [40], who have consistently reported that greater handgrip strength is strongly associated with a high BMI. This may be because boys with higher fat mass have more handgrip strength than girls despite the fact that there are no differences between the different weight status groups. Additionally, this relationship shows to be stable over the 4 years. Instead, a normal weight status was significantly associated with higher performance in cardiorespiratory fitness and vertical jump. The significance of cardiorespiratory fitness levels for cardiovascular health in the young population has been clearly demonstrated [41, 42]. Nevertheless, some research did not account for an important factor such as weight status in this association [43]. Thus, despite a positive relationship in handgrip strength with a high BMI as well as better cardiorespiratory fitness and muscular strength with an optimal BMI, it is important to consider data about body composition parameters when examining relationships of physical fitness and health outcomes.

### MFR, handgrip strength-to-BMI, and physical fitness

BMI remains one of the most widely used measures of adiposity and weight status in the young population [11, 44]. However, this indicator does not discriminate between fat mass and fat-free mass [14] and also does not reflect fat distribution and accumulation [45]. Accordingly, the MFR and handgrip strength-to-BMI ratio might be more reliable by addressing several of the limitations of BMI. In the elderly population, the handgrip strength-to-BMI ratio has been suggested for diagnosing sarcopenia [46]. In addition, the MFR may be a potential indicator for type 2 diabetes, metabolic syndrome, and hypertension in adults [47–49]. In youth, although the evidence is scare, Steffl et al. have suggested that the handgrip strength-to-BMI ratio can be used to identify children who are at risk of sarcopenic obesity [21]. Similarly, preschool children demonstrated that a greater handgrip strength-to-BMI ratio was associated with lower fat mass and percentage of body fat [50]. To date, there have been few studies that compare how these two indicators are related to fitness and health features in children and adolescents in the same sample. Our findings show that the MFR and handgrip strength-to-BMI ratio in both sexes were significantly correlated with cardiorespiratory fitness and vertical jump, while the handgrip strength-to-BMI ratio also showed a correlation with handgrip strength. Furthermore, these indicators tend to be representative over the 4 years studied.

Although MFR is a more difficult indicator to calculate as it depends on anthropometric measurements and body composition assessments obtained from specific equipment, a decrease in MFR was related to an excessive reduction in muscle strength and power in the lower extremities [51]. In contrast, body fat is strongly and inversely associated with 20-mSRT performance in children [52, 53]. This evidence supports the crucial role of other anthropometric parameters not covered by the BMI that should be taken into account for assessing the performance on physical fitness tests as well as for the health of children and adolescents. Therefore, more studies with large population data on BMI, MFR, and handgrip strength-to-BMI ratio in children and adolescents are warranted to provide further evidence on whether weight status should be considered not only through the BMI but also through other anthropometric indicators.

Major strengths of this study were (1) the novelty of this research, which includes other anthropometric indicators in young people and their relationship with different physical fitness variables; (2) the relatively large sample of rural active children and adolescents (*n* = 2256) who were measured using a standardised procedure; and (3) the data obtained from this population in four consecutive years. The current study also has some limitations that need to be considered. Although four different years have been studied, the design of this study is cross-sectional and therefore limits the interference with regard to the casualty of the associations examined. Moreover, the level of daily physical activity and socio-economic status were not controlled for or taken into account and may influence the generalisability of the results. Finally, body composition was not measured with advanced assessments or high precision by imaging techniques or specialised equipment such as DEXA or magnetic resonance. Nevertheless, as it is a large sample, bioimpedance may be a feasible measurement, since it is relatively cheaper, easily applied, and without radiation [54]; therefore, bioimpedance may be a good tool for routine assessment of body composition in this population. Thus, we recognise this limitation and suggest future studies using other specific equipment to confirm our findings.

## Conclusions

This study shows that weight status taken from BMI as well as MFR and handgrip strength-to-BMI is important indicators for health that are significant in different physical fitness parameters. A normal weight status presented significant values in cardiorespiratory fitness and vertical jump regarding those who were overweight and obese. Moreover, both indicators were positively correlated with handgrip strength, cardiorespiratory fitness and vertical jump (handgrip strength-to-BMI), and cardiorespiratory fitness and vertical jump (MFR) in both sexes and over the 4 years. Therefore, even though previous research determined that body composition analyses can allow for the identification and diagnosis of weight status based on BMI, the data of the present study show that the use of other body composition measurements such as MFR or handgrip strength-to-BMI can serve as a tools for identifying relationships of the paediatric population with physical fitness.

## Data Availability

The data presented in this study are available on request from the corresponding author. The data are not publicly available because they belong to minors.

## Abbreviations

BMI: Body mass index
MFR: Muscle-to-fat ratio
WHtR: Waist-to-height ratio
ASMM: Appendicular skeletal muscle mass
ALPHA: Assessing Levels of Physical Activity
20-mSRT: 20-M Shuttle-Run Test
WHO: World Health Organization
VO2max: Maximal oxygen uptake
cm: Centimetres
pc: Percentile
